# Functional Characterization and Whole-Genome Analysis of an Aflatoxin-Degrading *Rhodococcus pyridinivorans* Strain

**DOI:** 10.3390/biology11050774

**Published:** 2022-05-19

**Authors:** Dun Deng, Jiahong Tang, Zhichang Liu, Zhimei Tian, Min Song, Yiyan Cui, Ting Rong, Huijie Lu, Miao Yu, Jinbao Li, Rui Pang, Xianyong Ma

**Affiliations:** 1 State Key Laboratory of Livestock and Poultry Breeding, Institute of Animal Science, Guangdong Academy of Agricultural Sciences, Guangzhou 510640, China; dengdun@gdaas.cn (D.D.); tangjiahong2022@163.com (J.T.); liuzhichang@gdaas.cn (Z.L.); tianzhimei@gdaas.cn (Z.T.); songmin@gdaas.cn (M.S.); cuiyiyan@gdaas.cn (Y.C.); rongting@gdaas.cn (T.R.); luhuijie@gdaas.cn (H.L.); yumiao@gdaas.cn (M.Y.); 2 Key Laboratory of Animal Nutrition and Feed Science in South China, Ministry of Agriculture and Rural Affairs, Guangzhou 510640, China; 3 Guangdong Provincial Key Laboratory of Animal Breeding and Nutrition, Guangzhou 510640, China; 4 Guangdong Engineering Technology Research Center of Animal Meat Quality and Safety Control and Evaluation, Guangzhou 510640, China; 5 Guangdong Provincial Key Laboratory of Microbial Safety and Health, State Key Laboratory of Applied Microbiology Southern China, Institute of Microbiology, Guangdong Academy of Science, Guangzhou 510070, China; 2016202040064@whu.edu.cn (J.L.); pr839@163.com (R.P.)

**Keywords:** *Rhodococcus pyridinivorans*, AFB_1_ degradation, whole-genome sequencing, comparative genomic analysis, proteome, AFB_1_-degrading enzymes

## Abstract

**Simple Summary:**

The microbiological degradation of AFB_1_ has been a promising approach to control AFB_1_ contamination. Here, we characterize a *Rhodococcus pyridinivorans* strain that can efficiently degrade AFB_1_. The AFB_1_-degrading capacity of this bacterial strain was characterized, and the completed genome was sequenced and analyzed. Further proteomic analyses of this strain identified a total of 723 proteins in an extracellular component that showed the strongest capacity to degrade AFB_1_ (degradation rate 83.7%). Multiple potential AFB_1_-degrading enzymes, and enzymes that are reported to respond to AFB_1_ treatment, have been identified accordingly. These findings provide a genomic, proteomic, and experimental approach for characterizing an efficient AFB_1_-degrading bacterial strain with great potential for use in the remediation of AFB_1_ contamination.

**Abstract:**

Aflatoxin B_1_ (AFB_1_) is one of the most toxic, naturally occurring carcinogen compounds and is produced by specific strains of fungi. Crop contamination with AFB_1_ can cause huge economic losses and serious health problems. Many studies have examined the microbiological degradation of AFB_1_, especially the use of efficient AFB_1_-degrading microorganisms, to control AFB_1_ contamination. Here, we reported the identification of a new *Rhodococcus pyridinivorans* strain (4-4) that can efficiently degrade AFB_1_ (degradation rate 84.9%). The extracellular component of this strain showed the strongest capacity to degrade AFB_1_ (degradation rate 83.7%). The effects of proteinase K, SDS, temperature, pH, incubation time, and AFB_1_ concentration on the AFB_1_ degradation ability of the extracellular component were investigated. We sequenced the complete genome of this strain, encoding 5246 protein-coding genes and 169 RNA genes on a circular chromosome and two plasmids. Comparative genomic analysis revealed high homology with other *Rhodococcus* strains with high AFB_1_-degradation ability. Further proteomic analyses of this strain identified a total of 723 proteins in the extracellular component, including multiple potential AFB_1_-degrading enzymes, along with enzymes that are reported to response to AFB_1_ treatment. Overall, the results demonstrate that *R. pyridinivorans* 4-4 would be an excellent candidate for the biodegradation and detoxification of AFB_1_ contamination.

## 1. Introduction

Aflatoxins (AFTs) are a class of mycotoxins produced by specific fungal strains, such as *Aspergillus flavus* and *A. parasiticus* [1,2]. AFTs are secondary metabolites produced during metabolic processes, and several different types have been identified [3]. Six types of AFTs have public health and agricultural significance: AFB_1_, AFG_1_, AFB_2_, AFG_2_, AFM_1_, and AFM_2_ [4]. Among the AFTs, AFB_1_ is the most toxic, and was classified as a human class I carcinogen by the Agency for Cancer Research (IARC) in 1993 [5]. AFB_1_ exhibits strong hepatotoxicity, one of two important factors closely related to the occurrence of primary liver cancer, as well as a strong immunosuppressive toxicity which can reduce the body’s resistance to secondary infections of bacteria, fungi, and parasites [6,7].

Biological strategies have been used to reduce AFB_1_ levels in contaminated foods. One strategy is to directly inoculate AFB_1_-degrading bacterial strains that can metabolically or physically interact with AFB_1_ in food substrates [8]. However, more studies have indicated that the AFB_1_-degradation ability of microbials was enzymatic; thus, using enzymes to degrade AFB_1_ was a promising approach [9,10,11,12,13]. Some enzymes have been found to degrade AFB_1_, including aflatoxin oxidase (AFO), F_420_H_2_ dependent reductase (FDR), Mn peroxidase (MnP), laccases, manganese peroxidase, bacillus aflatoxin-degrading enzyme, and aflatoxin-detoxifizyme (ADTZ) [14,15,16,17,18,19]. However, the cellular localizations of the enzymes that degrade AFB_1_ are very different in different microbials. For example, the component of the salt tolerant *Tetragenococcus halophilus* CGMCC 3792 that degraded AFB_1_ was mostly located in the intracellular [20]. In both *Pseudomonas putida* and *Rhodococcus erythropolis*, the supernatants and cell lysates could degrade AFB_1_ [13,21]. The supernatants of some bacteria had stronger AFB_1_ degradability than other components, such as in *Stenotrophomonas maltophilia*, *Stenotrophomonas* sp. CW117, and *microbial consortium* TADC7 [22,23,24]. The AFB_1_ degradation in different organisms, by different enzymes and different components of cell, indicate the diversity and complexity of AFB_1_ degradation mechanisms [25].

The application of omics technology provides an important technical means for revealing the diversity and complexity of AFB_1_ degradation mechanisms. For instance, genomics has previously been used to find the AFB_1_-degrading enzymes in *Bacillus licheniformis* BL-010 and *Aspergillus niger* RAF106 [26,27]. Transcriptomics can detect the expression of genes in cells, and this technology has been used to detect the changes of gene expression levels in many kinds of cells [28,29,30]. Proteomics methods have also been applied to study the potential of Zn in alleviating AFB_1_-induced cytotoxicity [31]. Proteomics, combined with metagenomics, elucidated the detoxification mechanisms of *Lactobacillus casei* against AFB_1_ [32]. Comprehensive transcriptomics and proteomics have also been used to analyze the modulation of Aflatoxin production by *A. flavus* on different substrates [33]. On the other hand, metabolomics is usually used to analyze the products of AFB_1_ degradation, or to analyze the metabolites of cells in response to AFB_1_ treatment [34,35,36].

Previous studies have shown AFB_1_ degrading potential in different *Rhodococcus* species [11,37,38]. In this study, we identified a *Rhodococcus pyridinivorans* strain with remarkable AFB_1_-degrading ability. The AFB_1_-degrading capacity of this bacterial strain was characterized, and the complete genome was sequenced and analyzed. Finally, the proteome of the most active component of this strain was analyzed. The aim of this study was to provide insights into the functional characterization and genomic features responsible for AFB_1_ degradation in *Rhodococcus* sp.

## 2. Materials and Methods

### 2.1. Bacterial Strains and Plasmids

The experimental strains, *R. pyridinivorans* 4-4 and *R.* *equi* 4-9, were isolated from medium using coumarin as the only carbon source. Soil samples near a warehouse storing grains were used for separation AFB_1_ degradate strains, in Guangzhou, China. The crops in the warehouse were contaminated with AFB_1_, thus the nearby soil samples stood a good chance of AFB_1_ contamination, and microorganisms that interact with AFB_1_ were likely to be found. The pEASY-Blunt E1 expression plasmid (AMP+), *E. coli* Transetta (DE3) competent cells (CamR), and Trans1-T1 phage-resistant competent cells were purchased from TransGen Biotechnology Co., Ltd., Beijing, China.

### 2.2. The Detection of AFB_1_ Degradation Ability

The pure bacteria cultures were adjusted to the concentration of 1 × 10^9^ cfu/mL in 980 μL sterilized LB medium and was mixed with 20 μL of 5 μg/mL AFB_1_ standard solution in a glass tube. The mixed solution was incubated at 37 °C for 24 h without light. After completion of the AFB_1_ degradation reaction, 5 mL of acetonitrile and water (acetonitrile: water = 84:16, volume ratio) and 1 mL of n-hexane were added to stop the reaction. After shaking and mixing, the solution was left to rest for 1 h. Next, 200 μL of the extracted AFB_1_ solution was filtered into an injection flask through a 0.22 μm polyvinylidene fluoride membrane and the AFB_1_ content was detected by high-performance liquid chromatography (HPLC). The chromatographic conditions were as follows: XBridgetM C18 (4.6 mm × 250 mm, 5 μm) column; sample volume was 20 μL; mobile phase was methanol:water:acetonitrile = 35:10:55 (volume ratio); flow rate was 1 mL/min; column temperature was 30 °C; excitation wavelength of fluorescence was 360 nm; emission wavelength was 440 nm. An amount of 980 μL of sterilized LB medium without bacteria was mixed with 20 μL of 5 μg/mL AFB_1_ standard solution and used as a negative control.

### 2.3. Detection of the AFB_1_-Degrading Activity of Different Bacterial Components

The bacterial strain was cultured into 20 mL of LB in a shaker at 37 °C and 170 rpm for 12 h. The bacterial culture was then divided into two tubes and centrifuged at 10,625 g, at 4 °C for 10 min. The supernatant of one tube was filtered by a 0.22 μm water filtration membrane to obtain the extracellular component (EC) sample. The separated cell biomass was re-suspended and washed in phosphate buffer (PBS) and centrifuged at 10,625 g, at 4 °C for 10 min. The supernatant was again removed after centrifugation. This step was repeated twice, and then 10 mL of PBS was added to re-suspend the active cell sample. The active cell samples in the other tube were obtained as described above and were subsequently treated by ultrasonic crushing in an ice bath for 15 min (400 W, running for 5 s, stopping for 5 s). Centrifugation was then carried out at 10,625 g, at 4 °C for 10 min. The supernatant was centrifuged and filtered by a 0.22 μm water filtration membrane to obtain the intracellular component (IC) sample. Finally, the precipitate was re-suspended, washed, centrifuged three times, at last the precipitate were re-suspended with 10 mL PBS to obtain the cell debris (CD) sample. From each component, 980 μL was mixed with 20 μL of 5 μg/mL AFB_1_ standard solution in a 20 mL glass tube, then placed in a 37 °C incubator and incubated for 24 h away from light. The method to detect residual AFB_1_ is described in Section 2.2.

The factors that impact AFB_1_ degradation by bacterial component EC were further performed as follow: Proteinase K (1 mg/mL) was added to the EC samples and incubated with the AFB_1_ standard solution at 37 °C for 1 h. Then, 1% sodium dodecyl sulfate (1% SDS, *w*/*v*) was added into EC samples and incubated with the AFB_1_ standard solution at 37 °C for 6 h. To test the effect of temperature on the AFB_1_ degradation rate, EC samples were incubated with AFB_1_ at different temperatures (4, 20, 30, 37, 50, and 60 °C). Buffers with different pH values were also tested (citrate buffer was adjusted from 4.0 to 6.0, phosphate buffer from 6.0 to 8.0, and Tri-HCl buffer from 8.0 to 9.0) to see the impact of pH values. To characterize the influences of AFB_1_ concentration and incubation time, AFB_1_ was diluted to end concentrations of 50, 100, 250, 500, and 1000 ng/mL. Then, under the conditions of optimal temperature and pH value, each concentration of AFB_1_ was incubated with EC samples for 6, 12, 24, 48, or 72 h. At the end of these reactions under the different conditions, AFB_1_ degradation was measured, as described in Section 2.2.

### 2.4. Whole-Genome Sequencing of R. pyridinivorans 4-4

Genomic DNA of *R. pyridinivorans* 4-4 was extracted using a genomic DNA extraction kit (Magen Biotech, Guangzhou, China) according to the manufacturer’s instructions. Genomic DNA quality and integrity were evaluated by 0.8% agarose gel electrophoresis, in which a 50 kb band was observed. A NanoDrop 2000 spectrophotometer (Thermo Fisher Scientific, Wilmington, NC, USA) and a Qubit 3.0 fluorometer (Thermo Fisher Scientific, Wilmington, NC, USA) were used to measure the DNA concentration and purity.

According to the manufacturers’ instructions for the Illumina Hiseq and PacBio Sequel platforms, two different genomic DNA libraries were built. In Illumina Hiseq pre-sequencing, genomic DNA was first cut into about 500 bp using a Covaris M220 sonicator (Covaris, Woburn, MA, USA). Genomic DNA libraries were then generated using the Ultra™ DNA Library Prep Kit for Illumina (NEB, Foster City, CA, USA), with end-repair, adaptor ligation, size selection, and product enrichment steps. The libraries were evaluated using an Agilent Bioanalyzer 2100 (Agilent Technologies, Palo Alto, CA, USA) and a Qubit 3.0 fluorometer. Sequencing was performed on the Illumina Hiseq platform by GENEWIZ Ltd. (Suzhou, China). DNA samples were cut into 10 kb using a Covaris g-TUBE shearing device and were purified with AMPure XP Beads (Beckman Coulter, Pasadena, CA, USA) in the pre-processing of PacBio Sequel sequencing. The pre-processed genomic DNA was then constructed into a library using the PacBio SMRTbell library preparation kit, and the libraries were selected using BluePippin (Sage Science, Beverly, MA, USA). Finally, the libraries were sequenced using the PacBio Sequel platform.

### 2.5. Genome Assembly and Annotation

Cutadapt software was used to filter out low-quality reads from Illumina sequencing [39]. The hierarchical genome assembly process 4 (HGAP4) pipeline (Pacific Biosciences, SMRT Link 5.0) was used to perform the assembly of the PacBio reads. The filtered Illumina reads were compared with de novo assembled contigs using the Burrows–Wheeler Alignment software [40]. The alignment results were sorted using Picard (http://broadinstitute.github.io/picard/) released 8 April 2022, followed by base quality recalibration with Pilon. The final contigs were circularized by Circlator version 1.5.5 [41], and the completeness of the genomic data was assessed by BUSCO [42].

Gene annotation was conducted using Prokka v1.11 [43]. The protein-coding genetic annotation were further conducted using other public databases, including Swiss-Prot, NR (Non-redundant), COG (Clusters of Orthologous Groups) [44], Pfam [45], GO (Gene Ontology), and KEGG (Kyoto Encyclopedia of Genes and Genomes).

### 2.6. Comparative Genomic Analysis

For comparative genomic analysis, we downloaded the genome sequences of multiple *Rhodococcus* strains that were previously reported to degrade AFB_1_ with different efficiencies (Supplementary Table S1) [11,37,38]. The pan-genome of these *Rhodococcus* strains was estimated using Roary v3.11.2 software by using the Prokka output as the input [46], with a BLASTP identity cutoff of 60%. The alignment sequences of core genes from the analyzed *Rhodococcus* strains were used for subsequent phylogenetic analysis. The IQ-TREE v2.0.3 software was used to construct the maximum-likelihood (ML) tree, with threshold of “-m MFP” with 1000 ultrafast bootstrap replicates [47].

### 2.7. Liquid Chromatography Tandem Mass Spectrometry (LC-MS/MS) Analysis of Extracellular Component

The protein of the extracellular component was collected for proteomic analysis. Protein was extracted by acetone and dissolved by lysate under the ultrasound condition of (100 W, 0.8 s on, 0.8 s off, ultrasound 4 times, repeat twice). After the processes of 0.5 M TCEP and 55 mM MMTS solution, the protein was diluted in 8 M urea with 0.1 M Tris-HCl, followed by protein digestion with trypsin according to the FASP protocol [48]. The digested peptide was dissolved in the sample solution (0.1% formic acid, 2% acetonitrile). After being centrifuged, the supernatant was identified through mass spectrometry. The mass spectrum conditions were as follows: (a) Chromatographic column, 300 μm, idx 5 mm; Acclaim PepMap RSLC C18, 5 μm, 100 Å (Thermo, 160454); Acclaim PepMap 75 um × 150 mm, C18, 3 um, 100A (Thermo, 160321). (b) Mobile phase: mobile phase A, 0.1% formic acid; mobile phase B, 0.1% formic acid, 80% ACN; flow rate, 300 nL/min. (c) Analysis time: 120 min. The mass spectrometer (Thermo Scientific Q Exactive) was set with the following parameters: (a) first order mass spectrometry parameters: resolution, 70,000; AGC target, 3 × 10^6^; maximum IT, 100 ms; scan range, from 350 to 1800 m/z. (b) Second order mass spectrometry parameters: resolution, 17,500; AGC target, 5 × 10^4^; maximum IT, 120 ms; TopN, 20; NCE/stepped NCE, 27.

### 2.8. Protein Spectrum Data Analysis

The mass spectrometry data were used on MASCOT V2.8.01 (http://www.matrixscience.com/) to retrieve the UniProt database. The retrieval parameters were as follows: fixed modifications, carbamidomethyl (C); variable modifications, oxidation (M); enzyme, trypsin; maximum missed cleavages, 1; peptide mass tolerance, 20 ppm; fragment mass tolerance, 0.6 Da; mass values, monoisotopic; peptide expected value threshold, 0.05. The retrieved proteins were then aligned to the whole-protein sequences of *R. pyridinivorans* 4-4 generated by Prokka.

### 2.9. Data Statistics

The AFB_1_ degradation rate in the treated samples was counted according to the following equation:

AFB_1_ degradation rate (%) = [AFB_1_ content (C) − AFB_1_ content (T)] ÷ AFB_1_ content (C) × 100%

where C is the untreated control and T is the treated sample.

The experimental data were analyzed and processed using GraphPad Prism 5.0 software. Three replicates were performed for all experiments, and one-way ANOVA was used to assess significance. Multiple comparisons were further assessed with Duncan’s multiple range test.

## 3. Results

### 3.1. AFB_1_ Degradation Ability of R. pyridinivorans 4-4

**Figure 1:**
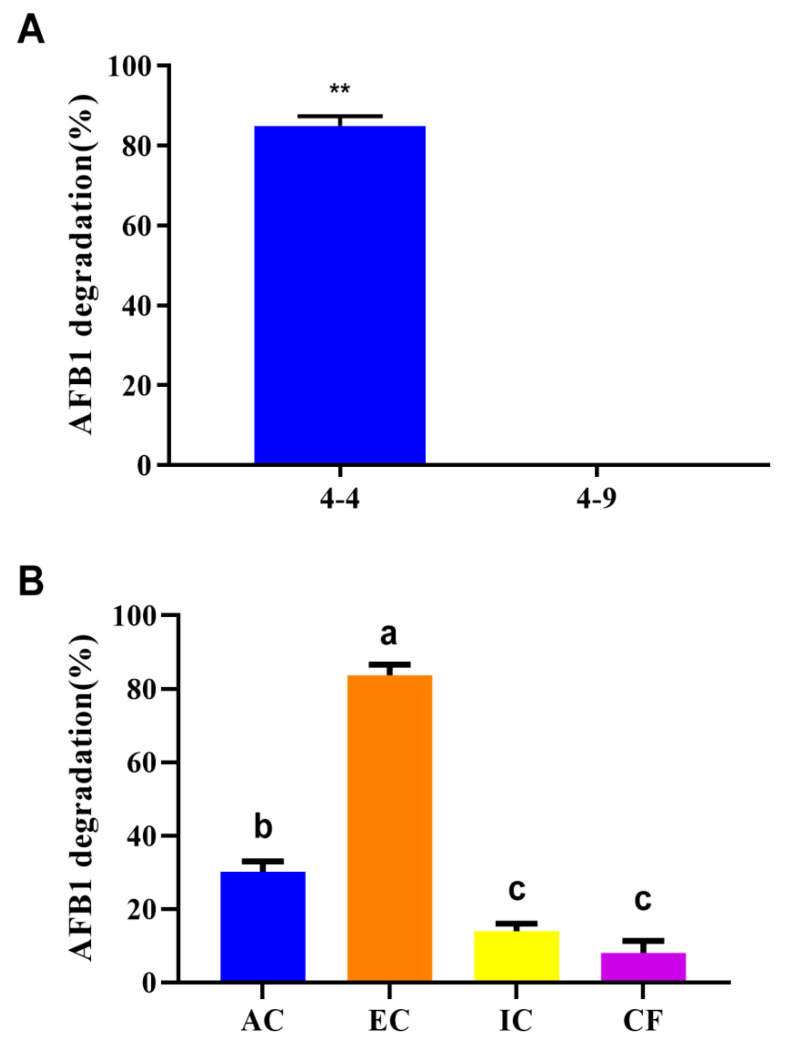
Characterization of the AFB_1_ degradation ability of *R. pyridinivorans* 4-4. (**A**) AFB_1_ degradation ability of *Rhodococcus* strains. (**B**) AFB_1_ degradation by different fractions of *R. pyridinivorans* 4-4. AC: active cell components; EC: extracellular component; IC: intracellular component; CF: cell fragments. Data are shown in means ± SEM. Significant (*p* < 0.01) difference between pairwise comparison is depicted by a double asterisk (one-way ANOVA). Values sharing the same letter are not significantly different at *p* < 0.05 (one-way ANOVA and Duncan’s multiple range test).

HPLC was used to determine the degradation rate of AFB_1_ by comparing the content of AFB_1_ before and after incubation with bacteria (Supplementary Figure S1). The AFB_1_ degradation rate of *R. pyridinivorans* 4-4 was significantly higher (84.9%) than that of the reference strain (*R. equi* 4-9, 0%) (*p* < 0.01, one-way ANOVA, Figure 1A). To characterize the source of the AFB_1_ degradation activity, cell fractionation was performed to obtain active cell components (AC), extracellular components (EC), intracellular components (IC), and cell fragments (CF). The EC had the highest AFB_1_ degradation rate (83.7%), followed by AC (30.2%), with lower degradation seen for IC and CF (13.9% and 8.0%, respectively) (Figure 1B). These results indicated that *R. pyridinivorans* 4-4 had a strong capacity to degrade AFB_1_, and its major active ingredients were within the extracellular fraction.

**Figure 2:**
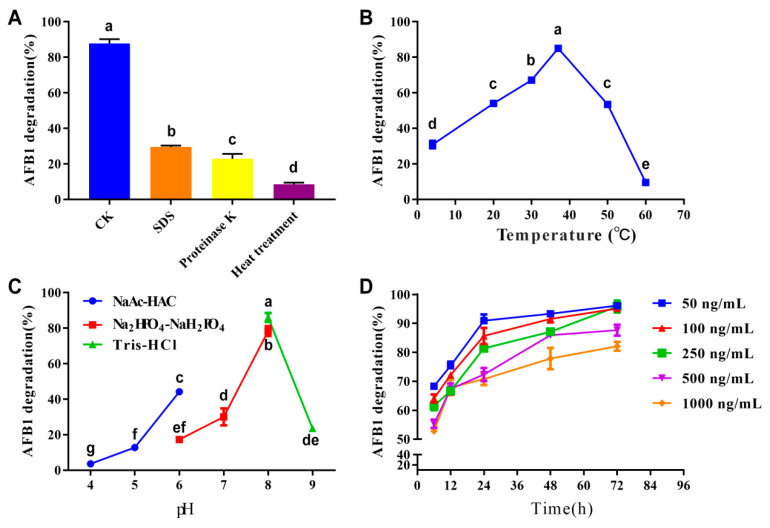
Effects of different treatment methods (**A**), temperature (**B**), pH conditions (**C**), and treated concentrations and times (**D**) on the AFB_1_ degradation rate of extracellular component fractions of *R. pyridinivorans* 4-4. CK in panel (**A**) refers to the control samples without any treatment. Data are shown in means ± SEM. Values sharing the same letter are not significantly different at *p* < 0.05 (one-way ANOVA and Duncan’s multiple range test).

The effects of proteinase K, SDS, temperature, pH, incubation time, and AFB_1_ concentration on AFB_1_ degradation ability of EC were investigated next. Treatment with SDS and proteinase K decreased the degradation by 58.2% and 65%, respectively, compared to the control (*p* < 0.05, one-way ANOVA, Figure 2A). The AFB_1_ degradation rate was also affected by temperature and pH. The optimal temperature was 37 °C (Figure 2B). When the temperature increased from 4 °C to 37 °C the degradation rate of AFB_1_ increased from 30.8% to 85.1%. However, when the temperature increased from 37 °C to 60 °C the degradation rate of AFB_1_ decreased continuously and was lowest (9.6%) at 60 °C. In Tris-HCl buffer, the optimal pH value was eight and the AFB_1_ degradation rate decreased with the increase of pH value. The degradation rate of AFB_1_ increased with pH in the citrate buffer (from 4 to 6) and the phosphate buffer (from 6 to 8) (Figure 2C). Incubation time and AFB_1_ concentration also affected the AFB_1_ degradation ability of EC. The degradation rate of AFB_1_ increased rapidly when the incubation time was less than 24 h. However, when incubation time was more than 24 h, the degradation rate of AFB_1_ increased slowly. Decreased AFB_1_ concentration increased the AFB_1_ degradation rate of EC when the concentration of AFB_1_ ranged from 50 ng/mL to 1000 ng/mL (Figure 2D).

### 3.2. Genomic Analysis of R. pyridinivorans 4-4

**Figure 3:**
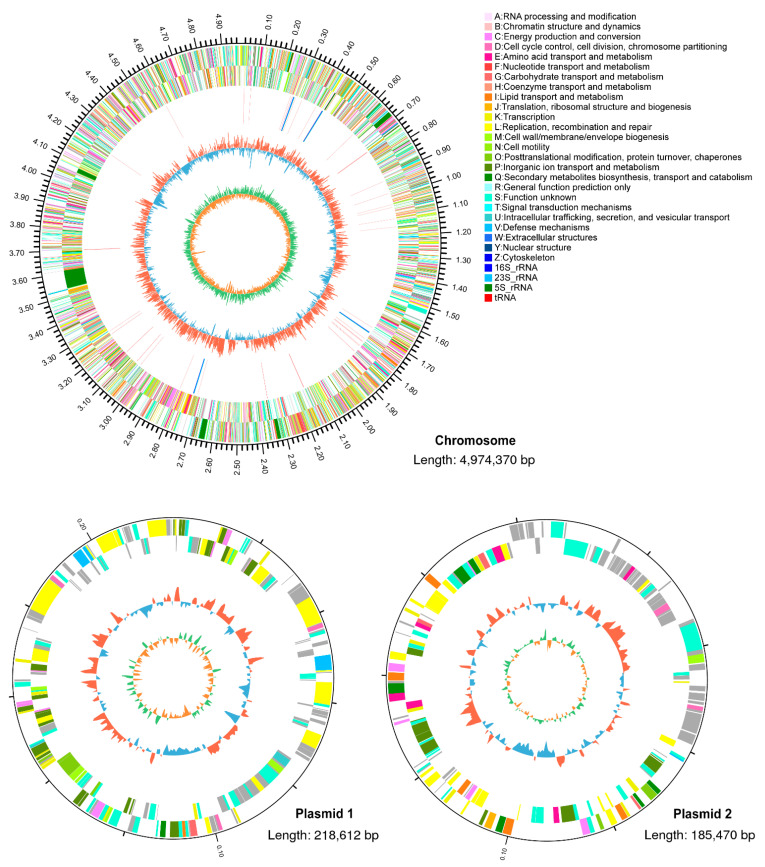
The whole genome of *R. pyridinivorans* 4-4. Circular representation of the genome with genomic annotation. The outer two rings (rings 1 and 2) represent the annotated genes, encoding proteins on the plus and minus strands, respectively. Different colors represent different COG categories for the corresponding genes. Ring 3 indicates non-coding RNAs. Ring 4 indicates the GC content (%), and ring 5 depicts the GC skew.

*R. pyridinivorans* 4-4 was sequenced to explore the genomic features responsible for AFB_1_ degradation. After assembly, we obtained the complete genome sequence of *R. pyridinivorans* 4-4, which comprised a chromosome and two plasmids. The chromosome was 4,974,370 bp in length, with an average GC content of 67.99%. The two plasmids were 218,612 bp and 185,470 bp in length, with average GC contents of 65.58% and 64.66%, respectively. A total of 5246 protein-coding genes, 12 rRNAs, 38 sRNAs, and 119 tRNAs were predicted from the complete genome of *R. pyridinivorans* 4-4 (Figure 3).

The results of the protein annotation statistics are presented in Table 1. A total of 4308 proteins were annotated to at least one COG functional category (Supplementary Figure S2). Most of the functional annotated proteins were associated with translation, amino acid transport and metabolism, energy production and conversion, lipid transport and metabolism, and inorganic ion transport and metabolism. GO annotation indicated that most of these genes were assigned to the functional categories of metabolic processes, cellular processes, membrane, catalytic activity, and binding (Supplementary Figure S3). These functional categories indicate that this bacterial strain may have a strong metabolic capacity.

### 3.3. Comparative Genomic Analysis of R. pyridinivorans 4-4 and other Rhodococcus Strains

The genome of *R. pyridinivorans* 4-4 was compared with the genomes of 31 other *Rhodococcus* strains, whose AFB_1_ degradation efficiencies were reported in previous studies (Supplementary Table S1) [11,37,38]. The analysis revealed a pan-genome of *Rhodococcus* strains, consisting of 65,006 protein-coding genes. Within the pan-genome, 117 core genes (present in all genomes) were identified. Additionally, 22,994 accessory genes (present in some, but not all strains) and 41,895 unique genes (unique to an individual strain) were determined. The sequence of *R. pyridinivorans* 4-4 includes 384 unique genes.

**Figure 4:**
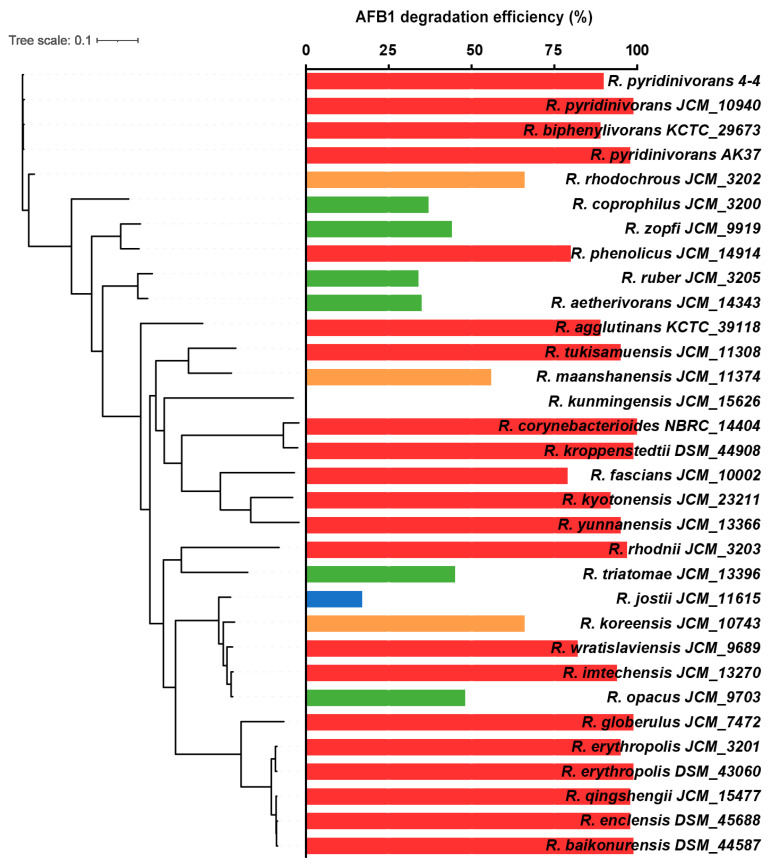
Maximum-likelihood phylogeny, estimated from the core gene alignments of 32 *Rhodococcus* strains. The AFB_1_ degradation efficiencies of these strains are shown. Rows in red indicate strong degrading ability.

The ML phylogenetic tree, constructed from core gene alignments, revealed genetic clustering of most of the strong AFB_1_-degrading (degradation rate >75%) *Rhodococcus* strains, with separation from strains with moderate (degradation rate within 50 and 75%) or low (degradation rate within 25 and 50%) AFB_1_ degradation (Figure 4). *R. pyridinivorans* 4-4 clustered with other two *R. pyridinivorans* strains and a single *R. biphenylivorans* strain, all with high AFB_1_ degradation ability. Additionally, two other clusters (cluster *R. globerulus-R. erythropolis-R. qingshengii-R. enclensis-R. baikonurensis* and cluster *R. corynebacterioides-R. kroppenstedtii-R. fascians-R. kyotonensis-R. yunnanensis*) also exhibited strong AFB_1_ degradation ability. These findings suggest that genus *Rhodococcus* is an important source of AFB_1_-degrading bacteria, with species *R. pyridinivorans* being of particular interest.

### 3.4. Proteomic Analysis of Extracellular Components

Considering that EC was the highest AFB_1_ degrading component of *R. pyridinivorans* 4-4, we analyzed the proteome of EC using the LC-MS/MS method. A total of 723 proteins were obtained from the proteomic analyses according to the mass spectrometry results (Supplementary Table S2). Among these proteins, many relevant AFB_1_-degrading enzymes, and enzymes that might respond to AFB_1_ treatment, were identified. These include enzymes such as NADPH-dependent aldo-keto reductases, serine protease, alkaline phosphatase, leucyl aminopeptidase, aminopeptidase, adenylate kinase, aldo-keto reductases, acetylcholinesterase, catalase, FDR, gamma-glutamyltransferase, gamma-glutamyl transpeptidase, NAD(P)H: quinine oxidoreductase, pyruvate kinase, and superoxide dismutase. In addition, many other oxidases, peroxidases, reductases, and oxidoredeuctases, have been identified (Supplementary Table S2). The existence of abundant enzymes with potential AFB_1_-degrading ability might elucidate the reason that the extracellular components can efficiently degrade the AFB_1_.

## 4. Discussion

Biological degradation of aflatoxin in foods and feeds by fungi and bacteria is considered a promising alternative to chemical degradation. In this study, we identified an *R. pyridinivorans* 4-4 strain with higher or faster AFB_1_ degradation ability than has previously been described for other bacteria or eukaryotes [9,24,25] (Figure 1A). In contrast, another *Rhodococcus* strain, *R. equi* 4-9, had almost no AFB_1_ degradation ability, indicating that the capacity to degrade AFB_1_ was not genus specific. To investigate which component accounted for the AFB_1_ degradation ability, we collected the active cell component (AC), extracellular component (EC), intracellular component (IC), and cell fragments (CF), and evaluated their AFB_1_ degradation ability. Although some studies reported that the intracellular component fraction of the salt-tolerant *Tetragenococcus halophilus* CGMCC 3792 and the cell-free supernatant and cell lysate of *Pseudomonas putida* reduced AFB_1_ effectively [13,20], our results showed the highest activity in the EC of *R. pyridinivorans* 4-4 (Figure 1B). This result was consistent with that of other studies [21,22].

Proteinase K and SDS reduced the degradation activity of *P. aeruginosa* N17-1 and *P. aeruginosa* M19 culture supernatants [20,49,50], and similarly reduced the activity of *R. pyridinivorans* 4-4 EC. AFB_1_ degradation ability was also affected by pH, temperature, incubation time, and the concentration of AFB_1_ (Figure 2). It has been reported that the buffer medium might have an impact on the protein activities of microorganisms [51,52]. In our results, the AFB_1_ degradation ability of the citrate and phosphate buffers were obviously different at a pH value of six (Figure 2C), indicating that the buffer medium might also affect the AFB_1_ degradation ability of EC. Moreover, while the concentrations of AFB_1_ ranged from 50 to 250 ng/mL, AFB_1_ degradation rates remained at a relatively high level incubation of 72 h (Figure 2D). This suggested that when AFB_1_ concentrations were less than 250 ng/mL, the AFB_1_ degradation enzymes in EC components were relatively sufficient, and that AFB_1_ could be adequately degraded in 72 h. However, when the AFB_1_ concentration was higher than 250 ng/mL (eg. 500 ng/mL and 1000 ng/mL), relatively lower degradation rates were observed as the enzyme contents became insufficient.

In order to understand the molecular basis of AFB_1_ degradation, we sequenced the whole genome of *R. pyridinivorans* 4-4 and then conducted a comparative genomic analysis. The COG and GO annotations suggest that this bacterial strain may have a strong metabolic capacity. The comparative genomic analysis indicated that *R. pyridinivorans* 4-4 clustered with *R. pyridinivorans* and *R. biphenylivorans* (Figure 4)—strains previously shown to have strong AFB_1_-degradation ability [37,38,53]. To further understand why the extracellular component has the highest AFB_1_ degradation efficiency, we analyzed the proteome of the extracellular components. The NADPH-dependent aldo-keto reductases, which are known to metabolize the AFB_1_ dihydrodiol by forming AFB_1_ dialcohol, were identified in the extracellular component of *R. pyridinivorans* 4-4 [54,55]. We also found serine proteases that can reversibly bind to AFB_1_ [56]. In addition, an FDR enzyme that has been reported to catalyze aflatoxin degradation was also found in the extracellular component of *R. pyridinivorans* 4-4 [14]. Furthermore, there were many enzymes previously known to respond to the AFB_1_ treatment in different conditions in the extracellular components. Alkaline phosphatase and superoxide dismutase are enzymes whose activity were elevated and reduced separately in AFB_1_-induced liver injury [57,58]. One study reported that, after ducks ate AFB_1_-contaminated maize, the activity of leucine aminopeptidase in the jejunum brush border increased [59]. In the study, leucyl aminopeptidase existed in the extracellular component. Gamma-glutamyltransferase is a well-identified enzyme whose content can be increased by AFB_1_ in animals and can catalyze the reactive intermediate of AFB_1_ [60,61,62,63]. Another interesting enzyme is NAD(P)H: quinone oxidoreductase, which increased in AFB_1_-treated livers, but its mRNA expressions in primary broiler hepatocytes (PBHs) were downregulated after AFB_1_ treatment [64,65]. Catalase undisputedly exists in the extracellular components, and studies have reported that its expression level and activity were lower when exposed to the AFB_1_ [66,67]. The expression level of glutamate dehydrogenase was also reduced when exposed to the AFB_1_ [68,69,70]. Cytochrome oxidase activities were decreased in liver mitochondria that were isolated from rats after AFB_1_ treatment [71]. Pyruvate kinase activity decreased in animals fed with a diet contaminated with AFB_1_ [72,73]. These enzymes, reported to respond to AFB_1,_ all exist in the extracellular components.

The structure of the AFB_1_ complex contains a functional phenol group, olefinic link, alcohol, ether, and ketone (PubChem CID 186907), and more the degradation of these groups mostly involves reduction-oxidation reaction. We speculate that AFB_1_ is not only easily oxidized by oxidase and peroxidase, but also reduced by reductase or catalyzed by oxidoreductase. Indeed, there are many oxidases, peroxidases, oxidoreductases, and reductases in the extracellular components (Supplementary Table S2). Thus, we hold the opinion that the AFB_1_-degrading ability of the extracellular components are not dependent on a single, or a few, components, but is the result of multiple components. However, to confirm this conjecture, more sophisticated experiments are needed in the future.

The optimal temperature for the EC of *R. pyridinivorans* 4-4 to degrade the AFB_1_ is 37 °C, and the optimal pH is eight (Figure 2B,C). Although the AFB_1_ degradation ability is low when the temperature is too low or too high and is easily affected by the value of pH, the optimal reaction conditions are well-suited for large-scale applications when compared to the conditions required for AFB_1_-degrading enzymes, such as *Pseudomonas* AFB_1_-degrading enzyme (PADE), which requires high temperature conditions that may not be suitable for practical applications [50]. Moreover, the EC is easy to obtain and has a high efficiency of AFB_1_ degradation. Therefore, it will be an efficient AFB_1_ degradation component. However, since the EC is a liquid medium, it is presently still unsuitable for direct application to remove AFB_1_ from food harvests and storage. Thus purifying these enzymes from EC and applying them to a practical production process might be a promising solution.

In conclusion, we characterized the AFB_1_ degradation ability of an efficient AFB_1_-degrading *R. pyridinivorans* strain. The extracellular component of this strain has the strongest AFB_1_ degradation, and the reaction conditions for the highest efficiency are mild and easy to achieve. We then obtained the whole genome sequence of this strain and conducted a comparative genomic analysis, which showed that this strain clustered into a cluster with strong AFB_1_ degradation ability. Based on proteomic analysis, we found multiple AFB_1_-degrading-relevant enzymes, which might account for the high AFB_1_-degrading ability of the extracellular components of *R. pyridinivorans* 4-4. Overall, the results demonstrate that *R. pyridinivorans* 4-4 has great potential for use in the remediation of AFB_1_ contamination.

## Figures and Tables

**Table 1:** Gene function annotation.

Database	Number of Annotated Genes	Percentage
Nr	4985	0.95
Swiss-Prot	3480	0.66
COG	4308	0.82
Pfam	3997	0.76
GO	3633	0.69
KEGG	2111	0.40
Overall	4986	0.95

## Data Availability

The whole-genome sequence generated in this study has been deposited in NCBI GenBank under BioProject PRJNA775846.

## Supplementary Materials

The following supporting information can be downloaded at https://www.mdpi.com/article/10.3390/biology11050774/s1: Figure S1. HPLC chromatogram of AFB1 degradation by *R. pyridinivorans* 4-4. Figure S2. Functional classification of the protein coding genes in *R. pyridinivorans* 4-4 (COG classification). Figure S3. Functional classification of the protein coding genes in *R. pyridinivorans* 4-4 (GO classification). Table S1. Information of *Rhodococcus* strains that were previously reported to degrade AFB_1_ with different efficiencies. Table S2. Identified proteins in the extracellular components of *R. pyridinivorans* 4-4.
