# Supplementary figures and images for: Functional Characterization and Whole-Genome Analysis of an Aflatoxin-Degrading Rhodococcus pyridinivorans Strain
Source: Biology (Basel). 2022 May 19;11(5):774. doi: 10.3390/biology11050774 (PMC9138218; doi:10.3390/biology11050774)

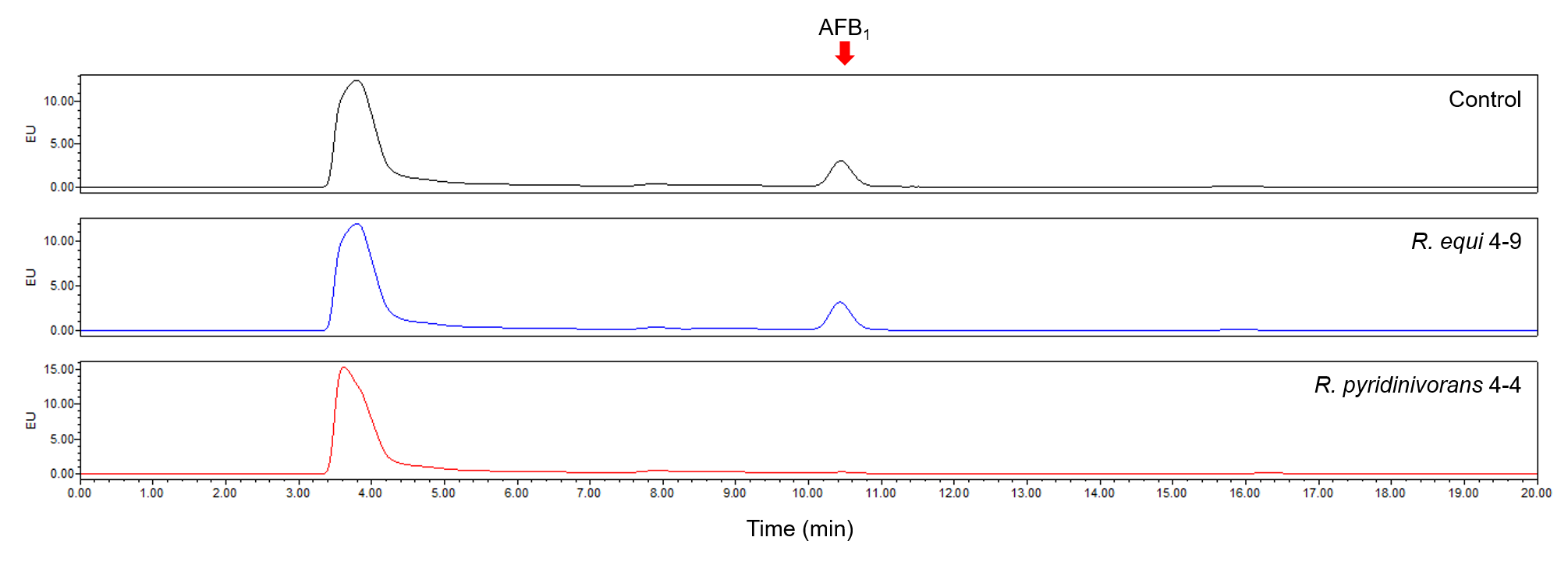

Supplement: Supplementary file 1 [file biology-11-00774-s001.zip › FigureS1.tif]

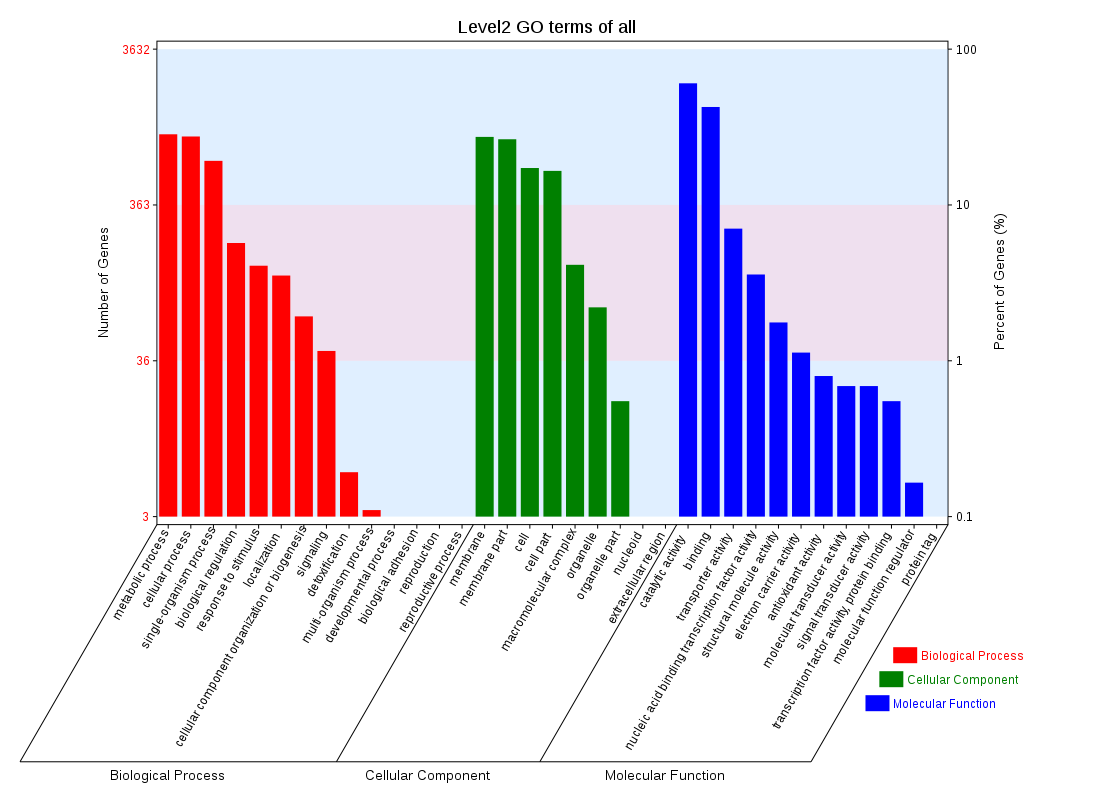

Supplement: Supplementary file 1 [file biology-11-00774-s001.zip › FigureS3.tif]

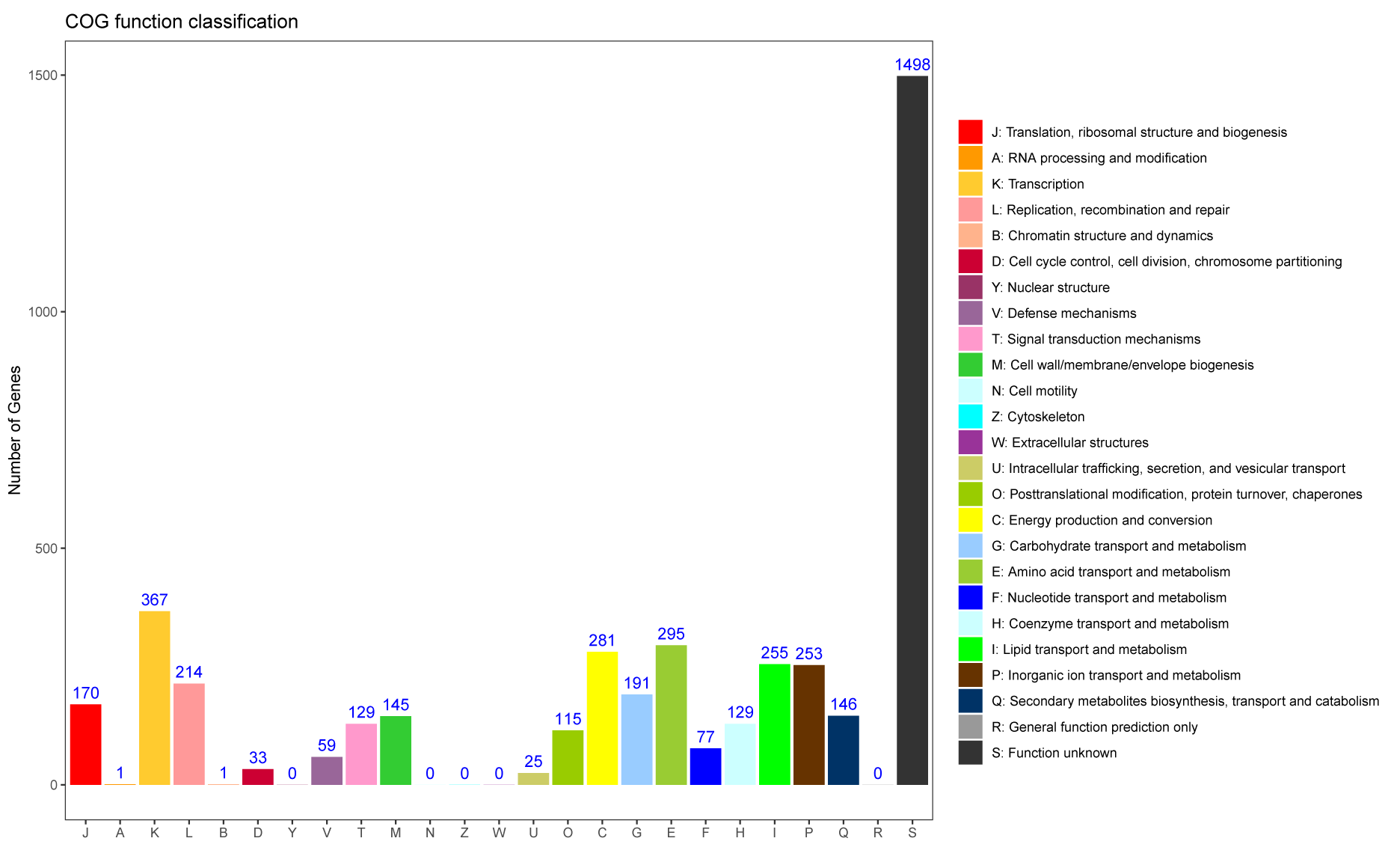

Supplement: Supplementary file 1 [file biology-11-00774-s001.zip › FigureS2.tif]
